# Glypican-3 Differentiates Intraductal Carcinoma and Paget’s Disease from Other Types of Breast Cancer

**DOI:** 10.3390/medicina59010086

**Published:** 2022-12-30

**Authors:** Fatemah OFO Alshammari, Anas O. Satari, Ahmed S. Aljabali, Yanal S. Al-mahdy, Yasmeen J. Alabdallat, Yahya M. Al-sarayra, Mohammad A. Alkhojah, Abdel rahman M. Alwardat, Mansour Haddad, Sameeh A. Al-sarayreh, Yousef M. Al-saraireh

**Affiliations:** 1 Department of Medical Laboratory Technology, Faculty of Health Sciences, The Public Authority for Applied Education and Training, Shuwaikh, Kuwait City 15432, Kuwait; 2 Faculty of Medicine, Mutah University, P.O. Box 7, Al-Karak 61710, Jordan; 3 Faculty of Medicine, Jordan University of Science and Technology, Irbid 22110, Jordan; 4 Faculty of Medicine, Hashemite University, Zarqa 13133, Jordan; 5 Al-Karak Governmental Hospital, Ministry of Health, Al-Karak 11118, Jordan; 6 Department of Internal Medicine, King Abdullah University Hospital, Irbid 22110, Jordan; 7 Faculty of Pharmacy, Yarmouk University, Irbid 21163, Jordan; 8 Department of Biochemistry and Molecular Biology, Faculty of Medicine, Mutah University, P.O. Box 7, Al-Karak 61710, Jordan; 9 Department of Pharmacology, Faculty of Medicine, Mutah University, P.O. Box 7, Al-Karak 61710, Jordan

**Keywords:** biomarker, breast cancer, glypican-3, immunohistochemistry, prognosis

## Abstract

*Background and Objectives*: breast cancer remains the most common health burden affecting females worldwide. Despite developments in breast cancer diagnostic approaches and treatment strategies, the clinical management of metastatic breast cancer remains challenging. Thus, there is a need to identify new biomarkers and novel drug targets for breast cancer diagnosis and therapy. Recently, aberrant glypican-3 (GPC3) expression in cancers has gained considerable interest in cancer research. The studies, however, have yielded contradictory results about GPC3 expression in breast cancer. Therefore, the current study aims to analyse GPC3 expression across a large panel of different breast cancer subtypes. *Materials and Methods*: GPC3 expression was immunohistochemically evaluated in 230 breast cancer patients along with eight normal tissues and its associations to clinical and demographic characteristics, as well as immunohistochemical biomarkers for breast cancer. Moreover, a public database consisting of breast cancer patients’ survival data and GPC3 gene expression information was used to assess the prognostic value of GPC3 in the survival of breast cancer patients. *Results*: GPC3 expression was only characterised in 7.5% of different histological breast cancer subtypes. None of the normal breast tissues displayed GPC3 expression. Interestingly, all cases of Paget’s disease, as well as 42.9% of intraductal and 16.7% of mucinous carcinomas were found to have GPC3 expression, where it was able to significantly discriminate Paget’s disease and intraductal carcinoma from other breast cancer subtypes. Importantly, GPC3 expression was found more often in tumours that tested positive for the expression of hormone receptors and human epidermal growth factor receptor 2 (HER2), indicating more favourable histological subtypes of breast cancer. Consequently, longer relapse-free survival (RFS) was significantly correlated with higher GPC3 mRNA expression. *Conclusions*: Our study proposes that GPC3 is a promising breast cancer subtype-specific biomarker. Moreover, GPC3 may have the potential to be a molecular target for the development of new therapeutics for specific subtypes of breast cancer.

## 1. Introduction

Breast cancer has a significant global influence on women’s health since it is the most common cancer in women worldwide, accounting for 24.2% of all cancers related to women. It has a mortality rate of 6.6% of the global cancer disease burden. Reported deaths attributed to breast cancer exceeded 15% globally in 2018. About one-third of breast cancer deaths are due to metastasis, particularly brain metastasis [1]. Despite recent improvements in treating breast cancer, challenges remain regarding accurate diagnostic procedures and treatment alternatives [2]. Therefore, novel biomarkers for early breast cancer detection, as well as new therapeutic strategies to better control metastatic breast cancer, are desperately needed.

Biomarkers are vital for early detection and effective management of breast cancer patients. They serve as tools for early diagnosis and provide accurate prognosis and timely predication for breast cancer patients during treatment. The discovery, development, and optimisation of diagnostic biomarkers that can improve breast cancer prognosis and therapeutic outcomes is accelerated by early detection of the disease. Development of breast cancer biomarkers may arise from tumour-associated macromolecules such as proteins, nucleic acid (DNA/RNA), and intact cells [3]. Modern molecular and artificial intelligence technologies have aided in discovering a wide variety of biomarkers that may have potential for breast cancer diagnosis, prognosis, and therapeutic implications. For instance, the presence of various circulating micro-RNAs in breast cancer offers a novel opportunity for the development of biomarkers for better diagnosis and accurate prognosis [4]. Moreover, machine learning and other artificial intelligence technologies have led to the identification of many dysregulated molecular molecules in breast cancer that have potential for the development of biomarkers. These include—but are not limited to—MAF1, ZC3H11A, VAX2, and ZFP91 [5,6]. Additionally, several novel therapeutic-based targeting agents, such as ESR1 DNA mutations, oligonucleotide analogs, CDK4/6, and poly (ADP-ribose) polymerase (PARP), have been identified and are under clinical validation [3].

Glypicans are membrane-bound heparan sulphate proteoglycans that are anchored to glycosylphosphatidylinositol. These are cell membrane glycoproteins in which chains of heparan sulphate glycosaminoglycan are attached to a protein core on a membrane-proximal region [7,8]. In mammals, the glypican family comprises six members, namely glypican-1 to 6 (GPC1 to GPC6). Glypicans can serve as co-receptors for a variety of signalling molecules that regulate cell proliferation, motility, and differentiation. Recent research indicates that individual glypican-specific biological activity is determined by its structure and by the growth factors mainly existing in its cellular environment [7,9]. Because of their unique structure, glypicans can interact differently with a wide range of protein classes. These include—but are not restricted to—growth factors, adhesion molecules, cytokines, chemokines, and extracellular matrix proteins [7,8]. Hence, it is not surprising that alterations in glypican expression are linked to a variety of human cancers.

One member of the glypican family that has attracted the interest of cancer researchers is GPC3. GPC3 is predominantly expressed during embryogenesis in a variety of different tissues. However, its expression disappears after birth, except in certain regenerating tissues such as mesothelium and the epithelia of the ovaries and breasts [10,11]. Surprisingly, GPC3 has primarily been explored in relation to cancer biology and human malignancies. GPC3 mutations and abnormal protein expression have been linked to tissue-specific cancers. Based on tissue type, GPC3 may serve as an oncofetal protein or a tumour-suppressor protein. GPC3 expression seems to be enhanced in cancers arising from tissues in which GPC3 expression is normally silenced, while its expression is inhibited in cancers derived from normal tissues that typically express GPC3. Several studies have investigated the expression of GPC3 in many cancers [10,11]. In this regard, overexpression of GPC3 was found in hepatocellular carcinoma, yolk sac tumours, clear cell ovarian carcinoma, and embryonal cancers, including neuroblastoma, Wilms’ tumour, and hepatoblastoma. Conversely, GPC3 expression was diminished in lung adenocarcinoma, mesothelioma, clear cell renal carcinoma, and ovarian and gastric cancers [10,11,12].

Currently, there is much debate regarding GPC3 expression in breast cancers. Limited studies have investigated GPC3 expression and its role in breast cancer progression. Recent evidence shows that GPC3 expression has been found in lower levels in breast cancer tissues compared to healthy normal tissues, although the studies were carried out in small sample sizes and focused on limited subtypes of breast cancer [13,14]. A similar pattern of expression was also demonstrated in other studies in certain subtypes of breast cancers, while other subtypes such as mucinous carcinoma showed a different mode of GPC3 expression [15,16]. Such heterogeneity in GPC3 expression between different subtypes of breast cancers is obvious and has manifested clearly in all studies examining GPC3 expression in breast cancer. On this basis, the current study aims to gain a better insight into GPC3 overexpression in a wide range of different subtypes of breast cancer and to correlate it with the expression of hormone receptors and other related molecules. Moreover, we utilised an updated version of an established database comprising gene expression data from breast cancer patients to assess whether the GPC3 expression has an impact on the survival rate of patients with breast cancer.

## 2. Materials and Methods

### 2.1. Tissue Specimens

Ethical approval was retrospectively obtained from the Institutional Review and Ethics Committee, Faculty of Medicine, University of Mutah (Reference No. 5222021 date: 23 February 2021). In addition, the Declaration of Helsinki’s principles were strictly followed in the study’s design and execution. Informed consent was not required for this study because of the exemption granted by the above-mentioned committee for the utilisation of paraffin-archived tissue samples of normal and breast cancer. During the five-year period (2015–2020), paraffin-embedded tissue samples from breast cancer patients and normal subjects were collected from King Hussein Medical Hospital, Royal Medical Services, Amman and King Abdullah University Hospital, Irbid, Jordan. All samples used in this study were for patients that had neither radiotherapy nor chemotherapy before the biopsy. The study comprised 250 patients of different subtypes of breast cancer and 8 normal tissues as controls. All related data about the study participants were extracted from the patients’ files, including age, tumour histological subtype, histological grade, tumour size, the status of lymph node metastasis, the status of distant metastasis, and the expression status of androgen receptors (AR), progesterone receptors (PR), oestrogen receptors (ER), epidermal growth factor receptors (EGFR), human epidermal growth factor receptor 2 (HER2), Ki67, and P53. All personal and clinical data about study participants were considered confidential and kept anonymous. 

### 2.2. Immunohistochemistry

After being dewaxed in xylene, the tissue sections were rehydrated in descending concentrations of alcohol and water. Following that, the tissue sections were incubated in 3% H_2_O_2_ for five minutes to inhibit intrinsic peroxidase activity. Next, the tissue sections were heated in a microwave at 650 W in citrate buffer (pH 6.0) for 20 min. To prevent nonspecific binding, the slides were then treated with normal goat serum at a concentration of 2.5%. After that, the slides were incubated for one hour with GPC3 rabbit monoclonal antibodies (concentration: 5 µg/mL, clone: SP86, Abcam, UK) at room temperature. After primary antibody incubation, the slides were thoroughly washed in a buffer and incubated with a polymer of peroxidase-conjugated goat anti-rabbit IgG for 30 min (MP-7451, Vector Laboratories, Burlingame, CA, USA). To develop immunoreactivity, diaminobenzidine chromogen solution (DAB) (Vector Laboratories Ltd., Peterborough, UK) was used for 3–5 min at room temperature. Following a wash in distilled water, the slides were counterstained with haematoxylin, dehydrated with increasing concentrations of alcohol, and then mounted using a DPX mounting medium. To confirm primary antibody specificity, hepatocellular carcinoma samples were used as a type of positive control. As a type of negative control, the primary antibody step was replaced with normal goat serum. For confirmation of primary antibody specificity, the GPC3 antibody was incubated with a blocking peptide for one hour. The slides were then incubated with the mixture instead of the primary antibody step. A comparison of the staining was then carried out between the slides incubated with the blocked primary antibody and the original primary antibody. A Leica DMRB microscope (Leica DMRB, Wetzlar, Germany) was used to evaluate immunoreactivity and a JVC video camera was used to capture images (Synoptics, Cambridge, UK).

### 2.3. Scoring

Three independent pathologists have manually and semiquantitatively analysed GPC3 expression using the Allred scoring system [17]. This unique system accounts for the percentage of stained cells as well as staining intensity. There were five categories for the percentage of stained cells. A score of 1 was assigned to tissue samples with staining of less than 1%. Tissue samples with staining between 2% and 10% received a score of 2. A score of 3 was assigned to tissue samples with staining between 11% and 33%. Tissue samples with staining between 34% and 66% scored 4. Finally, tissue samples with staining between 67% and 100% were allocated a score of 5. Regarding the staining intensity, four categories were utilised. A score of 0 was given for samples showing negative expression. Weak expression was given a score of 1. A score of 2 was allocated for samples showing moderate expression. Finally, a score of 3 was given for samples that showed strong expression. The total score was calculated by adding the score of the staining percentage to the score of the staining intensity. Accordingly, a total score out of eight categories was created. Negative GPC3 expression was defined as a score of ≤2, whereas positive GPC3 expression was allocated to samples with scores ranging from 3–8.

### 2.4. Analysis of GPC3 mRNA Expression and Survival of Breast Cancer Patients

The GPC3 mRNA expression data of 1085 patients with invasive breast carcinoma and 291 normal tissue samples were downloaded from The Cancer Genome Atlas database. The difference in GPC3 mRNA expression between invasive breast carcinoma tissues and healthy normal breast tissues was conducted using GEPIA (Gene Expression Profiling Interactive Analysis, http://gepia.cancer-pku.cn/index.html) (accessed on 20 November 2022) [18]. The mRNAseq data for TCGA invasive breast carcinoma were matched with normal TCGA and GTEx data. These data were transformed into transcripts per million (TPM) values, and the expression difference using boxplot analysis was performed using log2 (TPM+1) values. To assess the prognostic value of GPC3 in the survival of patients with breast cancer, the Kaplan–Meier Plotter online tool was utilised [14]. This tool, which is a publicly accessible database on kmplot.com, is based on gene expression data from TCGA, the European Genome-Phenome Archive (EGA), and the Gene Expression Omnibus (GEO). Presently, for breast cancer, the tool uses data for OS from 1879 patients and RFS from 4929 patients. Subsequently, data on the total number of patient cases with calculated median values for mRNA expression, a hazard ratio (HR) with 95% confidence intervals (CI), and log-rank-calculated *p*-values were obtained from the kmplot.com webpage (accessed on 25 November 2022). The statistical significance was defined as a log-rank *p*-value of ≤0.05.

### 2.5. Statistical Analysis

SPSS version 25 (IBM, Armonk, NY, USA) was used to analyse the available data, where variables were expressed as frequencies, and the multinomial goodness-of-fit test was used to identify differences between these variables. A *p*-value of ≤0.05 was deemed significan.

## 3. Results

### 3.1. Baseline Demographic and Clinical Features

Table 1 illustrates the baseline demographic and clinical features of 238 women who were involved in the study. The study included 230 cases of breast cancer of various histological subtypes and 8 cases of normal breast tissue. The mean age of the subjects included in the study was 48.3 ± 12.3 years. Of the subjects, 140 cases (58.8%) were aged less than or equal to 50 years, and 98 cases (41.2%) were aged more than 50 years. The study included 178 cases (74.8%) of invasive ductal carcinoma, 21 cases (8.8%) of invasive lobular carcinoma, 14 cases (5.9%) of intraductal carcinoma, 6 cases (2.5%) cases of mucinous adenocarcinoma, 11 cases (4.6%) of Paget’s disease, and 8 cases (3.4%) of normal breast tissue. The majority of cases (189 cases, 82.2%) were at grade II, while grade I and grade III consisted of 16 cases (7.0%) and 25 cases (10.8%), respectively. Most of the cases (139 cases, 60.4%) had a tumour size between 2 and 5 cm (T2), while 40 cases (17.4%) had a tumour size of less than 2 cm (T1); 33 cases (14.4%) had a tumour size of more than 5 cm (T3), and, finally, 18 cases (7.8%) had a tumour with direct extension to the chest wall (T4). Moreover, 66 cases (28.7%) tested positive for lymph node metastasis, while the others were free from it (164 cases, 71.3%). All cases tested negative for distant metastasis.

### 3.2. Prevalence of GPC3 Expression

**Figure 1:**
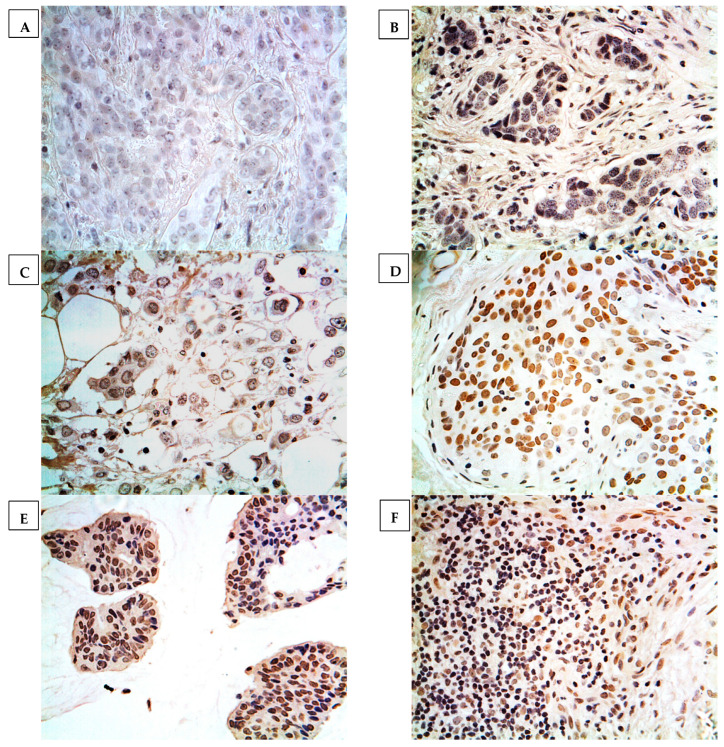
GPC3 expression in different histological subtypes of breast cancer. Classification was performed according to histopathological subtype. (**A**): normal breast tissue, (**B**): invasive ductal carcinoma, (**C**): invasive lobular carcinoma, (**D**): intraductal carcinoma, (**E**): mucinous adenocarcinoma, (**F**): Paget’s disease. Magnification ×400.

**Figure 2:**
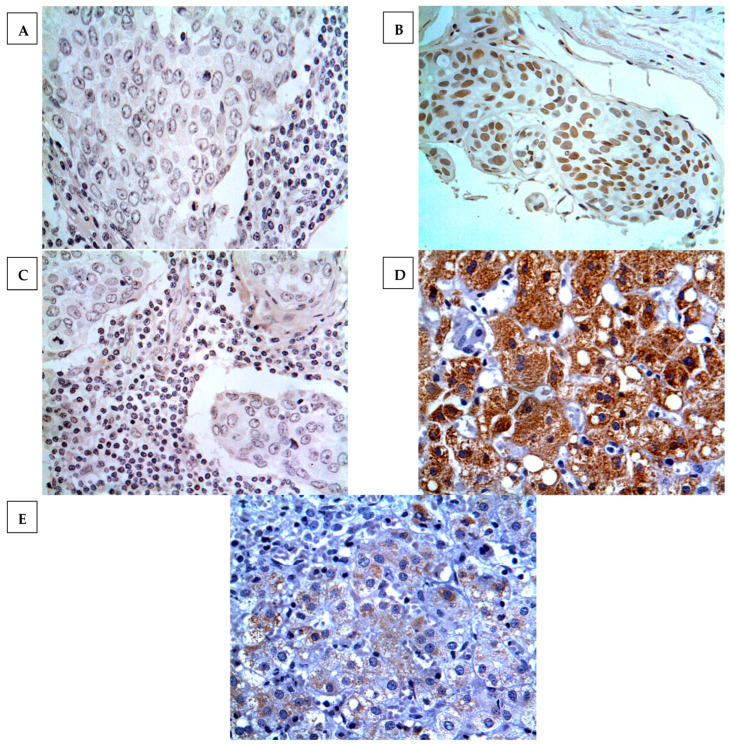
GPC3 expression in various types of experimental controls. (**A**) No GPC3 immunostaining was found in intraductal carcinoma tissue treated with normal goat serum instead of GPC3 primary antibody (negative control); (**B**) GPC3 expression was exhibited in intraductal carcinoma tissue incubated with GPC3 primary antibody; (**C**) no GPC3 expression was found in intraductal carcinoma tissue incubated with peptide-blocked primary antibody; (**D**) high GPC3 expression was displayed in hepatocellular carcinoma tissue treated with GPC3 primary antibody (positive control); and (**E**) weak GPC3 expression was determined in hepatocellular carcinoma tissue incubated with peptide-blocked primary antibody. Magnification (×400).

GPC3 expression was determined in 7.5% (18 cases) of breast cancers, where the expression was localised at the cell nucleus or cytoplasm. None of the normal breast cancer tissues showed positive expression, as displayed in Figure 1. Heterogeneous immunoreactivity was exhibited within the same tissue section and characterised as being low and with less intensity. Importantly, GPC3 expression was confirmed by using proper controls and inhibition experiments on immunoreactivity with the GPC3-blocked antibody. The results showed that negative controls exhibited no notable immunoreactivity, whereas strong and intense immunoreactivity was displayed in hepatocellular carcinoma tissues (positive control). Additionally, immunoreactivity was not determined in breast cancer specimens incubated with a blocked GPC3 antibody (Figure 2).

The results showed a highly significant correlation between GPC3 expression and the histological subtype of breast cancer (*p* = 0.001) (Table 1). GPC3 expression was only exhibited in Paget’s disease and intraductal and mucinous carcinomas, while other breast cancer subtypes displayed no expression at all. All cases of Paget’s disease showed GPC3 expression, while 42.9% (six cases) of intraductal and 16.7% (one case) of mucinous carcinomas displayed GPC3 expression. However, there was no correlation between GPC3 expression and age, grade, tumour size, status of lymph node metastasis, and status of distant metastasis.

Additional analysis was conducted on the associations between GPC3 expression and the expression of biomarkers, particularly hormone receptors (Table 2). The results showed significant findings where GPC3 was more frequently expressed in tumours that tested positive for hormone receptors AR (11.4%, 15 cases), ER (14.6%, 14 cases), and PR (11.4%, 13 cases). Similarly, frequent expression of GPC3 was found in tumours that were positive for EGFR (22.4%, 11 cases) and Ki67 (11.4%, 13 cases). Interestingly, all GPC3-positive cases in our study were also positive for HER2 expression. In contrast, GPC3 expression was found only in one case of tumours that tested positive for P53. It is surprising to note that all cases of Paget’s disease, which have GPC3-positive expression, were positive for all biomarker expressions except for P53.

### 3.3. The Expression of GPC3 Gene and Potential Prognostic Value

**Figure 3:**
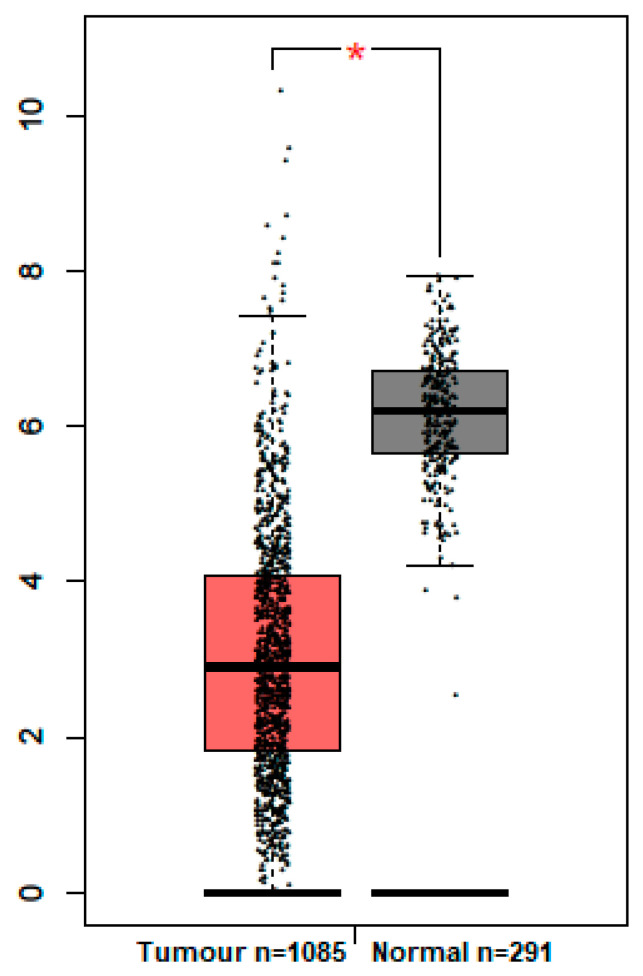
GPC3 mRNA expression in breast cancer versus normal healthy breast. * indicates a *p*-value of ≤0.05.

**Figure 4:**
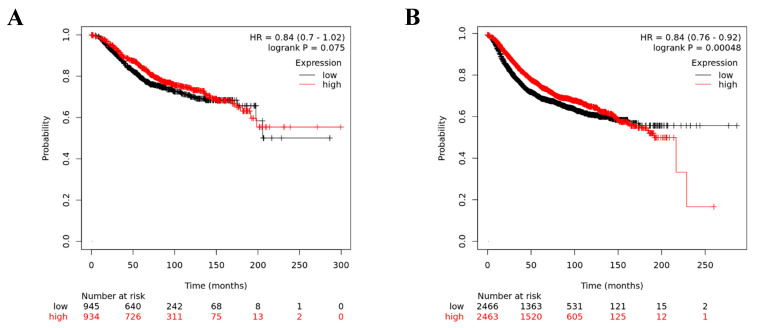
Kaplan–Meier survival curve of breast cancer patients based on GPC3 mRNA expression. (**A**) Overall survival (OS), and (**B**) relapse-free survival (RFS).

GEPIA analysis for GPC3 mRNA expression showed that significantly low levels of GPC3 mRNA were found in invasive breast carcinoma compared to matched normal tissues (Figure 3). To investigate whether GPC3 expression is associated to breast cancer patient prognosis, the Kaplan–Meier Plotter online tool was used to assess GPC3’s prognostic value in breast cancer. It was found that GPC3 mRNA expression had no significant association with patients’ OS of breast cancers (*p* = 0.075) (Figure 4A). However, patients with high GPC3 mRNA expression had significantly prolonged RFS compared to patients with low GPC3 mRNA expression (*p* = 0.00048) (Figure 4B).

## 4. Discussion

Breast cancer remains a serious global issue and a significant challenge for healthcare systems, with the majority of patients already suffering from metastatic breast cancer at the time of initial diagnosis. This metastatic disease is considered the leading cause of mortality among breast cancer patients [19]. Several molecular mechanisms allowing breast cancer to spread distally include—but are not limited to—aberrant expression of proteins such as cytochromes and chemokines, increased expression of matrix-degrading enzymes, and alterations in cytoskeletal function [20,21,22,23,24,25,26,27]. Many types of genes and proteins are being studied for their impact on metastasis, and one interesting molecule that has emerged from recent research is GPC3.

Here, we investigated the expression of GPC3 at the mRNA level in the data of invasive breast carcinoma matched with normal tissue samples obtained from TCGA by GEPIA mRNA analysis. The results demonstrated that a significantly lower level of GPC3 expression was found in invasive breast carcinomas compared to matched normal tissues. These results are consistent with our immunohistochemical findings, which show a similar pattern of GPC3 protein expression in some breast cancer histological subtypes, particularly invasive ones. In other words, almost all of the invasive breast carcinoma samples in our cohort were negative for GPC3 protein expression. Unfortunately, no TCGA data are available for GPC3 mRNA expression in some breast cancer subtypes showing GPC3 protein expression in our cohort, such as Paget’s disease and intraductal and mucinous carcinomas (see below for further details). Consistently, Moek et al. reported that GPC3 mRNA expression was identified in less than 14% of breast cancer samples examined, although no normal tissue samples were included in the analysis [16]. Similar results were also obtained in another study demonstrating that breast cancer samples displayed relatively lower levels of GPC3 mRNA compared to normal tissue samples [13]. However, these results should be interpreted with caution, as mRNA expression does not necessarily indicate protein translation or the protein might not be translocated to the cell cytoplasm or membrane. Additionally, another pitfall is that the target heterogeneity cannot be determined through mRNA profiling, as it is unable to distinguish the origin of target overexpression between cancer and noncancer cells. Therefore, an immunohistochemistry technique was used to characterise GPC3 protein expression in a wide spectrum of breast cancers.

By using immunohistochemistry, we successfully characterised GPC3 protein expression in different breast cancer subtypes of Paget’s disease and intraductal and mucinous carcinomas. Other breast cancer histological subtypes showed a lack of GPC3 protein expression. This is a novel finding, as GPC3 expression was able to significantly differentiate Paget’s disease and intraductal carcinoma from other breast cancer histological subtypes. Such a finding has neither been determined nor reported in other studies examining the GPC3 protein expression in breast cancer subtypes. Our results are also consistent with previous studies showing weak or absent GPC3 protein expression in other breast cancer subtypes [13,14,15,16,28]. Baumhoer et al. reported that GPC3 expression was weakly identified only in 17% of medullary breast carcinomas, 20% of lobular breast carcinomas, and 15% of mucinous carcinomas [15]. Moreover, similar results were reported by two other studies demonstrating GPC3 expression in 13% and 17% of breast cancer samples [13,16]. A possible explanation for these results came from two studies reporting the absence or weak expression of GPC3 in certain breast cancer subtypes due to the silencing of GPC3 by GPC3 promoter hypermethylation [14,28]. This downregulation of GPC3 was more predominant in patients who were negative for hormone receptors [28]. Such a finding was evident in the heterogeneity of GPC3 expression among different and certain breast cancer histological subtypes [13,15,16]. In other words, some breast cancer subtypes show GPC3 expression, such as Paget’s disease and intraductal and mucinous carcinomas, whereas other subtypes show no expression at all, such as invasive ductal carcinomas. This heterogeneity in GPC3 expression was clearly manifested in our cohort study and across many studies, and is proposed as a characteristic event in specific subtypes of breast cancers [13,16].

It was a surprising finding that the relative GPC3 protein expression was almost undetectable in patients lacking the hormone receptors AR, ER, and PR. A proposed mechanism for this finding has been reported before, where downregulation of GPC3 expression was determined in breast cancers because of the GPC3 promoter hypermethylation [28,29]. Such a mechanism was more evident and predominant in patients lacking hormone receptors [28]. A similar pattern of GPC3 downregulation was also detected in other tumours, such as mesotheliomas and ovarian cancer [29]. Moreover, our results for the levels of GPC3 expression in patients who were negative for hormone receptors were comparable with results obtained by other studies [13,16]. Additionally, it is interesting to note that almost all GPC3-positive cases of Paget’s disease and intraductal carcinomas were positive for all hormone and HER2 receptors. This indicates that GPC3 may be preferentially expressed in patients with more favourable histological subtypes of breast cancer. Moreover, several significant correlations between GPC3 expression and EGFR, HER2, P53, and Ki67 were detected. Low levels of GPC3 expression were determined in all tumours that were positive for EGFR, HER2, P53, and Ki67. A comparable pattern of GPC3 expression was found in patients who were positive for EGFR, HER2, and Ki67 [13,16]. Overall, these results encourage further research investigating GPC3 expression and its relation to hormone receptors in certain breast cancer subtypes.

Our survival analysis showed that high levels of GPC3 mRNA expression were significantly associated with longer RFS. The positive impact on survival is consistent with the findings of several other studies, which have shown that GPC3 may have a unique protective function in the development of human breast cancer [29,30,31]. Upregulation of GPC3 was shown to decrease cell proliferation in almost all breast cancer cell lines examined [14]. Furthermore, in a murine model, GPC3 expression was shown to suppress metastasis of breast cancer cells by reducing cell proliferation, survival, and motility. Importantly, GPC3 expression was found to promote mesenchymal–epithelial transition (EMT) by inhibiting autocrine and paracrine activities of canonical Wnt. This led breast cancer cells to change the organisation of their cytoskeleton, reduce their capability to migrate and clone, become more sensitive to apoptosis, and become less invasive [29]. Moreover, GPC3 ectopic expression has led to increased susceptibility of breast cancer cells to apoptosis induced by increased serum depletion [32]. However, another study found that neither GPC3 protein nor mRNA expression was associated with RFS [13]. These results can be explained by the small sample size used in the study, and the authors themselves proposed conducting a study with a larger patient cohort. Generally, these findings support the assumption that GPC3 may play a role as a tumour suppressor in the development of breast cancer. Therefore, considering GPC3 gene expression as a breast cancer subtype-specific biomarker may aid in assessing breast cancer prognosis.

Concordant with the above surprising results, GPC3 pathway analysis was performed using the Kyoto Encyclopedia of Genes and Genomes (KEGG) pathway analysis [33]. It was found that GPC3 is connected with the regulation of cell proliferation, plasma membrane, and extracellular space. This is in line with previous studies performed on other types of cancers [34,35]. Moreover, several coexpressed genes were identified in the correlation network for GPC3 and its coexpressed genes using gene ontology (GO)/KEGG pathway analysis. These genes’ GO (gene ontology) analyses revealed that they were enriched in processes related to cell adhesion, angiogenesis, and inflammatory. Furthermore, KEGG analysis revealed that these genes were associated with a number of biological processes, primarily in the Ras, Rap1, PI3K-Akt, and chemokine signalling pathways. This agrees with previous studies performed on other cancer types such as pancreatic ductal adenocarcinoma and hepatocellular carcinoma [34,35].

Because metastasis is the most important factor affecting the survival of breast cancer patients, discovering new molecules that can revert or promote EMT is critical for the development of novel cancer therapies [29]. Even though GPC3 expression was found in less than 20% of the breast cancer subtypes examined in general, this may be relevant to the growing use of personalised therapy. This is particularly exemplified by Paget’s disease and intraductal carcinomas overexpressing GPC3 in our study. For these aggressive breast cancer histological subtypes, targeted therapy strategies are lacking [36]. Glypicans are interesting cancer targets, and the latest developments in immunotherapy targeting GPC3 in cancer have shown great promise in clinical trials. GPC3 has been investigated in treatments for HCC, and clinical trials with a monoclonal antibody targeting GPC3 have demonstrated good tolerability [37]. Furthermore, a GPC3 vaccine has been found to infiltrate tumour tissue in paediatric tumours, and the antibody has been shown to stimulate solid tumour regression [38]. These findings for immunotherapeutics targeting GPC3 in different cancers show that GPC3 is a promising target to develop novel cancer therapies, and further research into the therapeutic potential in particular breast cancer subtypes will be of great value.

## 5. Conclusions

The current study demonstrated promising results where GPC3 expression significantly differentiated Paget’s disease and intraductal carcinomas from other breast cancer histological subtypes. Almost all other breast cancer histological subtypes showed no expression at all. Moreover, GPC3 expression was mainly displayed in tumours that were positive for hormone and HER2 receptors. This means that GPC3 was frequently expressed in patients with more favourable histological subtypes of breast cancer. Importantly, GPC3 mRNA expression was revealed as a strong prognostic predictor of breast cancer patients. Elevated levels of GPC3 mRNA expression connoted longer RFS. Moreover, GPC3 was found to be coexpressed with different genes responsible for several biological roles, including cell adhesion, angiogenesis, and inflammation. Consequently, we propose the use of GPC3 as a diagnostic biomarker for identifying some of breast cancer-specific histological subtypes such as Paget’s disease and intraductal carcinomas. Moreover, GPC3 may potentially serve as a selective target for development of novel therapeutics for particular subtypes of breast cancer expressing GPC3.

## Figures and Tables

**Table 1:** Baseline demographic and clinical features of breast cancer patients.

	GPC3 Expression		
Characteristic:	Negative n = 220 (92.5%)	Positive n = 18 (7.5%)	*p* Value
Age:			
≤50 (n = 140, 58.8%)	128 (91.4%)	12 (8.6%)	0.482
>50 (n = 98, 41.2%)	92 (93.9%)	6 (6.1%)	
Histological subtype:			
Invasive ductal carcinoma (n = 178, 74.8%)	178 (100.0%)	0 (0.0%)	0.001
Invasive lobular carcinoma (n = 21, 8.8%)	21 (100.0%)	0 (0.0%)	
Intraductal carcinoma (n = 14, 5.9%)	8 (57.1%)	6 (42.9%)	
Mucinous adenocarcinoma (n = 6, 2.5%)	5 (83.3%)	1 (16.7%)	
Paget’s disease (n = 11, 4.6%)	0 (0.0%)	11 (100.0%)	
Normal (n = 8, 3.4%)	8 (100.0%)	0 (0.0%)	
Histological grade:			
I (n = 16, 7.0%)	13 (81.3%)	3 (18.7%)	0.148
II (n = 189, 82.2%)	177 (93.7%)	12 (6.3%)	
III (n = 25, 10.8%)	22 (88.0%)	3 (12.0%)	
Tumour size:			
T1 (n = 40, 17.4%)	35 (83.3%)	7 (16.7%)	0.067
T2 (n = 139, 60.4%)	131 (95.6%)	6 (4.4%)	
T3 (n = 33, 14.4%)	30 (90.9%)	3 (9.1%)	
T4 (n = 18, 7.8%)	16 (88.9%)	2 (11.1%)	
Lymph node metastasis:			
Negative (n = 164, 71.3%)	151 (92.1%)	13 (7.9%)	0.929
Positive (n = 66, 28.7%)	61 (92.4%)	5 (7.6%)	
Distant metastasis:			
Negative (n = 230, 100.0%)	212 (92.2%)	18 (7.8%)	
Positive (n = 0, 0.0%)	0 (0.0%)	0 (0.0%)	N/A

**Table 2:** Association between GPC3 expression and immunohistochemical markers. androgen receptors (AR), progesterone receptors (PR), oestrogen receptors (ER), epidermal growth factor receptors (EGFR), human epidermal growth factor receptor 2 (HER2).

Immunohistochemical Markers	Negative n = 220 (92.5%)	Positive n = 18 (7.5%)	*p* Value
AR:			
Negative (n = 106, 44.5%)	103 (97.2%)	3 (2.8%)	
Positive (n = 132, 55.5%)	117 (88.6%)	15 (11.4%)	0.013
ER:			
Negative (n = 115, 48.3%)	111 (96.5%)	4 (3.5%)	
Positive (n = 123, 51.7%)	109 (85.4%)	14 (14.6%)	0.009
PR:			
Negative (n = 139, 58.4%)	134 (96.4%)	5 (3.6%)	
Positive (n = 99, 41.6%)	86 (88.6%)	13 (11.4%)	0.026
EGFR:			
Negative (n = 189, 79.4%)	182 (96.3%)	7 (3.7%)	
Positive (n = 49, 20.6%)	38 (77.6%)	11 (22.4%)	0.001
HER2:			
Negative (n = 72, 30.3%)	72 (100.0%)	0 (0.0%)	
Positive (n = 166 69.7%)	148 (89.2%)	18 (10.8%)	0.004
Ki67:			
Negative (n = 124, 52.1%)	119 (96.0%)	5 (5.0%)	
Positive (n = 114, 47.9%)	101 (88.6%)	13 (11.4%)	0.032
P53:			
Negative (76, 31.9%)	59 (77.7%)	17 (22.3%)	
Positive (n = 162, 68.1%)	161 (99.3%)	1 (0.7%)	0.013

## Data Availability

The data presented in this study are available on request from the corresponding author. The data are not publicly available due to privacy and ethical concerns.
